# Supplementary material for: Habitat loss weakens the positive relationship between grassland plant richness and above-ground biomass
Source: eLife. 2024 Mar 18;12:RP91193. doi: 10.7554/eLife.91193 (PMC10948147; doi:10.7554/eLife.91193)
Supplement: Supplementary file 1. [file elife-91193-supp1.docx]

**Supplementary file 1.** List of 130 species of vascular plants recorded across 130 sites in this study.

| Family | Genus | Species | Habitat specialisation |
| --- | --- | --- | --- |
| Amaranthaceae | *Amaranthus* | *Amaranthus blitum* | W |
| Amaranthaceae | *Chenopodium* | *Chenopodium aristatum* | W |
| Amaranthaceae | *Bassia* | *Bassia scoparia* | W |
| Amaranthaceae | *Chenopodium* | *Chenopodium glaucum* | W |
| Amaranthaceae | *Suaeda* | *Suaeda glauca* | S |
| Amaranthaceae | *Corispermum* | *Corispermum mongolicum* | S |
| Amaranthaceae | *Kochia* | *Kochia prostrata* | S |
| Amaranthaceae | *Bassia* | *Bassia dasyphylla* | S |
| Amaranthaceae | *Corispermum* | *Corispermum chinganicum* | S |
| Amaranthaceae | *Salsola* | *Salsola collina* | W |
| Amaryllidaceae | *Allium* | *Allium anisopodium* | S |
| Amaryllidaceae | *Allium* | *Allium polyrhizum* | S |
| Amaryllidaceae | *Allium* | *Allium mongolicum* | S |
| Amaryllidaceae | *Allium* | *Allium bidentatum* | S |
| Amaryllidaceae | *Allium* | *Allium tenuissimum* | S |
| Amaryllidaceae | *Allium* | *Allium ramosum* | S |
| Apiaceae | *Bupleurum* | *Bupleurum scorzonerifolium* | S |
| Apiaceae | *Ferula* | *Ferula bungeana* | S |
| Apocynaceae | *Cynanchum* | *Cynanchum thesioides* | W |
| Asparagaceae | *Asparagus* | *Asparagus cochinchinensis* | S |
| Asteraceae | *Heteropappus* | *Heteropappus altaicus* | S |
| Asteraceae | *Artemisia* | *Artemisia argyi* | S |
| Asteraceae | *Xanthium* | *Xanthium sibiricum* | W |
| Asteraceae | *Saussurea* | *Saussurea amara* | S |
| Asteraceae | *Saussurea* | *Saussurea japonica* | S |
| Asteraceae | *Artemisia* | *Artemisia annua* | W |
| Asteraceae | *Leontopodium* | *Leontopodium leontopodioides* | S |
| Asteraceae | *Cirsium* | *Cirsium japonicum* | W |
| Asteraceae | *Ixeris* | *Ixeris polycephala* | W |
| Asteraceae | *Echinops* | *Echinops sphaerocephalus* | S |
| Asteraceae | *Artemisia* | *Artemisia frigida* | S |
| Asteraceae | *Serratula* | *Serratula centauroides* | S |
| Asteraceae | *Hemistepta* | *Hemistepta lyrata* | W |
| Asteraceae | *Hippolytia* | *Hippolytia trifida* | S |
| Asteraceae | *Taraxacum* | *Taraxacum mongolicum* | W |
| Asteraceae | *Echinops* | *Echinops gmelini* | S |
| Asteraceae | *Olgaea* | *Olgaea lomonossowii* | S |
| Asteraceae | *Ixeris* | *Ixeris gracilis* | W |
| Asteraceae | *Ixeridium* | *Ixeridium gracile* | W |
| Asteraceae | *Scorzonera* | *Scorzonera austriaca* | S |
| Asteraceae | *Artemisia* | *Artemisia halodendron* | S |
| Asteraceae | *Artemisia* | *Artemisia capillaris* | W |
| Asteraceae | *Neopallasia* | *Neopallasia pectinata* | S |
| Asteraceae | *Artemisia* | *Artemisia scoparia* | W |
| Asteraceae | *Filifolium* | *Filifolium sibiricum* | S |
| Boraginaceae | *Lappula* | *Lappula myosotis* | S |
| Brassicaceae | *Lepidium* | *Lepidium apetalum* | W |
| Brassicaceae | *Dontostemon* | *Dontostemon dentatus* | S |
| Brassicaceae | *Ptilotricum* | *Ptilotricum canescens* | S |
| Caryophyllaceae | *Gypsophila* | *Gypsophila davurica* | S |
| Caryophyllaceae | *Gypsophila* | *Gypsophila desertorum* | S |
| Caryophyllaceae | *Silene* | *Silene conoidea* | W |
| Caryophyllaceae | *Silene* | *Silene aprica* | S |
| Caryophyllaceae | *Gypsophila* | *Gypsophila licentiana* | S |
| Convolvulaceae | *Convolvulus* | *Convolvulus arvensis* | W |
| Convolvulaceae | *Convolvulus* | *Convolvulus ammannii* | S |
| Crassulaceae | *Orostachys* | *Orostachys fimbriatus* | S |
| Cyperaceae | *Carex* | *Carex korshinskyi* | S |
| Ephedraceae | *Ephedra* | *Ephedra sinica* | S |
| Euphorbiaceae | *Euphorbia* | *Euphorbia humifusa* | W |
| Euphorbiaceae | *Euphorbia* | *Euphorbia esula* | S |
| Fabaceae | *Astragalus* | *Astragalus scaberrimus* | S |
| Fabaceae | *Astragalus* | *Astragalus melilotoides* | S |
| Fabaceae | *Astragalus* | *Astragalus dahuricus* | S |
| Fabaceae | *Oxytropis* | *Oxytropis bicolor* | S |
| Fabaceae | *Oxytropis* | *Oxytropis diversifolia* | S |
| Fabaceae | *Lespedeza* | *Lespedeza bicolor* | S |
| Fabaceae | *Medicago* | *Medicago ruthenica* | S |
| Fabaceae | *Corethrodendron* | *Corethrodendron fruticosum var. mongolicum* | S |
| Fabaceae | *Gueldenstaedtia* | *Gueldenstaedtia verna* | S |
| Fabaceae | *Thermopsis* | *Thermopsis lanceolata* | S |
| Fabaceae | *Astragalus* | *Astragalus galactites* | S |
| Fabaceae | *Oxytropis* | *Oxytropis racemosa* | S |
| Fabaceae | *Oxytropis* | *Oxytropis leptophylla var. turbinata* | S |
| Fabaceae | *Astragalus* | *Astragalus adsurgens* | S |
| Fabaceae | *Lespedeza* | *Lespedeza daurica* | S |
| Gentianaceae | *Gentiana* | *Gentiana dahurica* | S |
| Gentianaceae | *Gentiana* | *Gentiana scabra* | S |
| Geraniaceae | *Erodium* | *Erodium stephanianum* | S |
| Iridaceae | *Iris* | *Iris lactea* | S |
| Iridaceae | *Iris* | *Iris lactea var. chinensis* | S |
| Iridaceae | *Iris* | *Iris tenuifolia* | S |
| Lamiaceae | *Dracocephalum* | *Dracocephalum heterophyllum* | S |
| Lamiaceae | *Thymus* | *Thymus mongolicus* | S |
| Lamiaceae | *Scutellaria* | *Scutellaria scordifolia* | S |
| Lamiaceae | *Phlomis* | *Phlomis umbrosa* | S |
| Lamiaceae | *Lagochilus* | *Lagochilus ilicifolius* | S |
| Lamiaceae | *Dracocephalum* | *Dracocephalum moldavica* | S |
| Lamiaceae | *Lagopsis* | *Lagopsis supina* | W |
| Linaceae | *Linum* | *Linum usitatissimum* | W |
| Nitrariaceae | *Peganum* | *Peganum harmala* | S |
| Orobanchaceae | *Cymbaria* | *Cymbaria dahurica* | S |
| Plantaginaceae | *Plantago* | *Plantago asiatica* | W |
| Plantaginaceae | *Plantago* | *Plantago depressa* | W |
| Plumbaginaceae | *Limonium* | *Limonium aureum* | S |
| Poaceae | *Echinochloa* | *Echinochloa crusgali* | W |
| Poaceae | *Cleistogenes* | *Cleistogenes hancei* | S |
| Poaceae | *Agropyron* | *Agropyron cristatum* | S |
| Poaceae | *Cleistogenes* | *Cleistogenes squarrosa* | S |
| Poaceae | *Stipa* | *Stipa breviflora* | S |
| Poaceae | *Setaria* | *Setaria viridis* | W |
| Poaceae | *Eragrostis* | *Eragrostis pilosa* | W |
| Poaceae | *Enneapogon* | *Enneapogon borealis* | S |
| Poaceae | *Alopecurus* | *Alopecurus aequalis* | W |
| Poaceae | *Phragmites* | *Phragmites australis* | W |
| Poaceae | *Elymus* | *Elymus dahuricus* | S |
| Poaceae | *Koeleria* | *Koeleria cristata* | S |
| Poaceae | *Bromus* | *Bromus inermis* | W |
| Poaceae | *Cleistogenes* | *Cleistogenes songorica* | S |
| Poaceae | *Leymus* | *Leymus chinensis* | S |
| Poaceae | *Stipa* | *Stipa capillata* | S |
| Poaceae | *Digitaria* | *Digitaria ischaemum* | W |
| Poaceae | *Stipa* | *Stipa grandis* | S |
| Polygonaceae | *Polygonum* | *Polygonum aviculare* | W |
| Polygonaceae | *Polygonum* | *Polygonum sibiricum* | W |
| Portulacaceae | *Portulaca* | *Portulaca oleracea* | W |
| Primulaceae | *Androsace* | *Androsace umbellata* | S |
| Primulaceae | *Lysimachia* | *Lysimachia barystachys* | W |
| Ranunculaceae | *Thalictrum* | *Thalictrum petaloideum* | S |
| Ranunculaceae | *Thalictrum* | *Thalictrum squarrosum* | S |
| Rosaceae | *Potentilla* | *Potentilla betonicifolia* | S |
| Rosaceae | *Chamaerhodos* | *Chamaerhodos erecta* | S |
| Rosaceae | *Potentilla* | *Potentilla bifurca* | W |
| Rosaceae | *Potentilla* | *Potentilla anserina* | W |
| Rosaceae | *Potentilla* | *Potentilla verticillaris* | S |
| Rosaceae | *Geum* | *Geum aleppicum* | W |
| Rosaceae | *Potentilla* | *Potentilla acaulis* | S |
| Rutaceae | *Haplophyllum* | *Haplophyllum dauricum* | S |
| Thymelaeaceae | *Stellera* | *Stellera chamaejasme* | S |
| Zygophyllaceae | *Tribulus* | *Tribulus terrester* | W |

Note: S: Grassland specialists; W: Weeds.
