# Supplementary material for: Habitat loss weakens the positive relationship between grassland plant richness and above-ground biomass
Source: eLife. 2024 Mar 18;12:RP91193. doi: 10.7554/eLife.91193 (PMC10948147; doi:10.7554/eLife.91193)
Supplement: Supplementary file 2. [file elife-91193-supp2.docx]

**Supplementary file 2.** Four optimal models of landscape context, environment factors, and plant diversity affecting above-ground biomass.

| Ranking | Model | R^2^ | AICc |
| --- | --- | --- | --- |
| 1 | AGB~HL+SWT+GSR+WR | 0.47** | 1213.7 |
| 2 | AGB~HL+FPS+SWT+GSR+WR | 0.47** | 1215.2 |
| 3 | AGB~HL+LST+SWT+GSR+WR | 0.47** | 1215.3 |
| 4 | AGB~FPS+SWT+GSR+WR | 0.46** | 1215.3 |

Note: AGB: above-ground biomass; HL: habitat loss; FPS: fragmentation per se; SWT: soil water content; LST: land surface temperature; GSR: grassland specialist richness; WR: weed richness; **: significance at the 0.01 level.
