# Supplementary material for: Habitat loss weakens the positive relationship between grassland plant richness and above-ground biomass
Source: eLife. 2024 Mar 18;12:RP91193. doi: 10.7554/eLife.91193 (PMC10948147; doi:10.7554/eLife.91193)
Supplement: Supplementary file 3. [file elife-91193-supp3.docx]

**Supplementary file 3.** Effects of interaction terms between habitat loss and fragmentation per se and plant richness on above-ground biomass.

| Response variable | Predictor variable | Standardised estimate |
| --- | --- | --- |
| AGB | SR | 0.65** |
|  | SR×HL | -0.23* |
|  | SR×FPS | -0.10 |

Note: AGB: above-ground biomass; SR: plant richness; HL: habitat loss; FPS: fragmentation per se; * and ** represent significance at the 0.05 and 0.01 levels, respectively.
