# Supplementary material for: Habitat loss weakens the positive relationship between grassland plant richness and above-ground biomass
Source: eLife. 2024 Mar 18;12:RP91193. doi: 10.7554/eLife.91193 (PMC10948147; doi:10.7554/eLife.91193)
Supplement: Supplementary file 4. [file elife-91193-supp4.docx]

**Supplementary file 4.** Summary of the principal component analysis for the four fragmentation indices.

| Results | PC1 |
| --- | --- |
| Eigenvalue | 2.04 |
| Proportion explained | 0.51 |
| Coefficient of the PD | -0.87 |
| Coefficient of the ED | -0.61 |
| Coefficient of the AREA_MN | 0.93 |
| Coefficient of the ENN | -0.17 |

Note: PD: patch density; ED: edge density; AREA_MN: mean patch area; ENN: mean nearest-neighbor distance; PC1: the first principal component of the four fragmentation indices.
