# Supplementary material for: Habitat loss weakens the positive relationship between grassland plant richness and above-ground biomass
Source: eLife. 2024 Mar 18;12:RP91193. doi: 10.7554/eLife.91193 (PMC10948147; doi:10.7554/eLife.91193)
Supplement: Supplementary file 5. [file elife-91193-supp5.docx]

**Supplementary file 5.** Variance inflation factors of predictor variables for above-ground biomass.

| Variable | Variance inflation factor |
| --- | --- |
| Habitat loss | 2.49 |
| Fragmentation per se | 2.09 |
| Grassland specialist richness | 1.30 |
| Weed richness | 1.15 |
| Soil water content | 1.09 |
| Land surface temperature | 1.29 |
